# Naming the “baby” or the “beast”? The importance of concepts and labels in healthcare safety investigation

**DOI:** 10.3389/fpubh.2023.1087268

**Published:** 2023-02-10

**Authors:** Siri Wiig, Carl Macrae, Jan Frich, Sina Furnes Øyri

**Affiliations:** ^1^Department of Quality and Health Technology, Faculty of Health Sciences, SHARE Centre for Resilience in Healthcare, University of Stavanger, Stavanger, Norway; ^2^Nottingham University Business School, University of Nottingham, Nottingham, United Kingdom; ^3^Department of Health Management and Health Economics, Faculty of Medicine, University of Oslo, Oslo, Norway; ^4^Division for Health Services, Norwegian Institute of Public Health, Oslo, Norway

**Keywords:** safety investigation, patient safety, inspection, investigation, learning

## Abstract

This paper focuses on concepts and labels used in investigation of adverse events in healthcare. The aim is to prompt critical reflection of how different stakeholders frame investigative activity in healthcare and to discuss the implications of the labels we use. We particularly draw attention to issues of investigative content, legal aspects, as well as possible barriers and facilitators to willingly participate, share knowledge, and achieve systemic learning. Our message about investigation concepts and labels is that they matter and influence the quality of investigation, and how these activities may contribute to system learning and change. This message is important for the research community, policy makers, healthcare practitioners, patients, and user representatives.

## 1. Introduction

Concepts and labels matter. The name we give to an activity can frame and shape how the activity is conducted, what it means to those engaged in it, and what consequences it might have. This is particularly important for responses and investigative activities that can follow patient safety events (1–6). Does it matter if the response to an event is being named as an accident investigation, inspection, exploration, analysis, case, complaint, inquiry—or even a prosecution? The differences between these labels and their connotations are not trivial. Even so, debates about terminology and labels are, perhaps surprisingly, rarely explicit in the field of patient safety.

In this paper, we aim to prompt critical reflection of how different stakeholders frame investigative activity in healthcare: What are the implications of the labels we use? We particularly draw attention to issues of investigative content, legal aspects, as well as possible barriers and facilitators to willingly participate, share knowledge, and achieve systemic learning.

## 2. What's the difference between concepts and content?

In healthcare systems around the world, a diverse range of organizations and processes may be involved when adverse safety events occur. Table 1 indicates the main Norwegian bodies and their role in response to adverse events. The Norwegian healthcare system is among the first to establish a national independent body to investigate safety events [the Norwegian Healthcare Investigation Board (NHIB)] as a supplement to established regulatory bodies (1, 4).

**Table 1:** Overview of main bodies from the Norwegian context and their role and mandate in following up of adverse events.

**Type of body**	**System level**	**Purpose for safety event follow up**	**Sanctioning power**	**Legal framework for governmental authority**	**Concept used for accident investigation activity**
Norwegian Board of Health Supervision (NBHS)	Organizational and individual scope. Subordinate to the Ministry of Health and Care Services	National, external inspection of the healthcare services performance and patient treatment in accordance with the regulatory principle of sound professional practice	(1) notification about breach of conduct, or (2) administrative sanctions against healthcare personnel and/or healthcare providers and organizations	Norwegian healthcare legislation Act relating to public supervision of health and care services (1984)	Regulatory inspection
County governors (CG)	Organizational and individual scope. Administratively subordinate to the Ministry of Local Government and Regional Development Under supervision of Norwegian Board of Health Supervision (NBHS)	Regional, external inspection of the healthcare services performance and patient treatment in accordance with the regulatory principle of sound professional practice	(1) Notification about breach of conduct, or (2) Administrative sanctions against healthcare personnel and/or healthcare providers and organizations	Norwegian healthcare legislation Act relating to public supervision of health and care services (1984)	Regulatory inspection
Norwegian Healthcare Investigation Board (NHIB)	System-wide scope. Subordinate to the Norwegian Ministry of Health and Care Services.	Independent, multi-level and multidisciplinary investigation, set to promote system-wide learning and patient safety.	Non-punitive, non-sanctioning authority.	Norwegian healthcare legislation Act on the Norwegian Healthcare Investigation Board (2017).	Exploration
The Norwegian System of Patient Injury Compensation	Individual scope. Subordinate to the Norwegian Ministry of Health and Care Services.	Handling of applications in compensation claims from patients. In cases of financial loss as a result of an injury caused by inadequate medical treatment, compensation will be granted (under specific conditions).	No sanctioning authority against healthcare personnel. In cases where the conditions are not met and compensation not granted, there may be an option of submitting an appeal to the National Office for Health Service Appeals.	Norwegian Tort Law and Non-Statutory Law Act on patient injury compensation (2001)	Case; claim
Law enforcement; criminal prosecution	Organizational and individual scope.	Investigation of cases where Norwegian law has been violated.	Criminal sanctions; penalties.	The Penal Code (2005) and other Norwegian laws.	Police investigation and criminal prosecution.

### 2.1. Regulatory inspection of adverse events

In Norway, regulatory bodies at regional (County Governors) and national (Norwegian Board of Health Supervision) level examine cases of reported patient harm, complaints, and severe adverse events in healthcare. A legal logic underpins the processes that involves assessing whether patients received treatment according to the regulatory principle of sound professional practice and guidelines. If not, sanctions can apply to individuals (warning, restrictions, withdraw license) and organizations (fines, warnings).

Investigations by the Norwegian Board of Health Supervision and the County Governors are often referred to as “inspections” [e.g., (7, 8)]. Healthcare professionals and organizations risk sanctions if they are involved in an adverse event where an inspection reveals a violation of law and regulations. This legal process of “inspection” has been in place for years, but the media still refers to these as “investigations.” Some regulatory inspections become high profile cases (9, 10) and the media tends to focus on individual patients, professionals, and managers. From the perspective of learning, information sharing and trust this erroneous labeling of regulatory inspections as “investigations” may be counterproductive: people may subsequently confuse the role and objectives of these regulatory inspections (which can carry significant legal jeopardy) with other types of investigative activities that are more oriented to learning and systems improvement.

### 2.2. Independent exploration of adverse events

The recently established Norwegian Healthcare Investigation Board (NHIB) conducts independent investigations of severe adverse events. NHIB decides which cases to investigate and how comprehensive these investigations should be. The purpose of NHIB activities is learning and improvement (4). Notably, the Norwegian concept used when referring to NHIB investigations translates in English to “exploration.” The legal framework also uses “explorations” as part of the title and mandate of NHIB. In contrast, the English translation of NHIB's name and title of the law both use the label “investigation.” The operationalization of the “exploration” that NHIB conducts is broad, system-wide, multidisciplinary, learning-focused, and does not carry risk of sanctions for the healthcare personnel or organizations involved. Naming these activities an “exploration” represents a strong framing effect signaling that this is a safe and exploratory process to participate in for professionals. Moreover, the term “exploration” is, in contrast to “investigation,” “inspection” or “inquiry,” a marker of a more open-minded, tentative, and formative process that accommodates the complex, interactive systems and networks of causality associated with healthcare safety.

## 3. Concluding remarks and recommendations

Ongoing debates within healthcare on the importance of independent safety investigations, regulatory inspections and legal enforcement highlight the importance of sharing information, facilitating learning, promoting a just culture, building trust and actively involving patients' and families after adverse events (1, 11–17). We believe there is also a need to reflect more systematically on how we name and frame the different activities that follow safety events, and the connotations of these labels in the public domain. Labels, and the concepts and principles they imply, can deeply influence how people interpret and engage with a process, how willing people are to share their knowledge and experiences, and what consequences people expect. We believe there is a need to more clearly articulate and explore the differences between what concepts, labels, and names mean in practice. Naming the “baby” ambiguously, or with a concept loaded with alternative meanings, may lead people to fear a “beast,” confusing or distracting them from efforts to share, learn and improve.

Ultimately, how concepts and labels are used in practice and interpreted by different groups is an empirical question. The issues highlighted here warrant close and critical investigation and would form the foundation for a productive research programme. From a more practical and clinical perspective, there are important opportunities for clinicians, patients, managers and regulators to engage in more critically reflective and collective examination of the concepts and labels that are routinely used in relation to adverse events; in particular, it would seem important to refine and clarify the language used by–and to describe the roles of–the different bodies involved after adverse events. Such collective deliberation should not simply be focused in relation to an individual specific adverse event, but should be part of a broader endeavor to develop and improve the systems in place to learn from both disruptive conditions and normal situations.

For organizations and individuals to learn, information must be openly and honestly shared and used in good faith for the purposes of improvement. This can be particularly challenging when clinical staff are exposed to external or supervisory bodies entering the clinical field to collect information about adverse events. As such, it is critically important to carefully design spaces and processes that can enable sharing and learning. At the same time, there is a need to acknowledge the potential limitations and tensions inherent in the processes of external review of adverse events, particularly if those bodies have sanctioning powers, and also if they have the ability to disclose events or information that may risk identifying healthcare staff, patients or organizations, even if particular information characteristics are secured and anonymity is regulated by law.

Overall, based on the arguments advanced here, we recommend that policymakers, regulators, practitioners, media outlets and the research community need to engage in a careful exploration of how language, concepts and labels can deeply support–or impede–the processes they describe. We propose making a terminological shift in the labeling of regulatory and supervisory activities that are aimed at learning and quality improvement, shifting to a language centered on 'systemic exploration'. Such an approach may signal sensitivity to the importance of building public, professional and patient trust and accommodating the complexity and networks of causality associated with adverse events in healthcare.

## Data availability statement

The original contributions presented in the study are included in the article/supplementary material, further inquiries can be directed to the corresponding author.

## Author contributions

SW had the idea and developed the first draft of the article which was further developed in close collaboration with SØ, CM, and JF. All authors contributed to the revision and have approved the final version of the article.
